# (*Z*)-3β-(2-Chloro­anilino)-17(20)-pregnene

**DOI:** 10.1107/S1600536813007290

**Published:** 2013-04-05

**Authors:** Jun-yi Hu, Jiang Wang, Ying-qian Xu, Yun Gao, Guo-yong Xiao

**Affiliations:** a Center of Separation Technology, School of Chemical Engineering, University of Science and Technology Liaoning, Anshan 114051, People’s Republic of China

## Abstract

In the pregnene fragment of the title compound, C_27_H_38_ClN, the three six-membered rings exhibit chair conformations and the five-membered ring has a distorted envelope form with the fused C atom not bearing a methyl group as the flap atom. The amino group is involved in the formation of an intra­molecular N—H⋯Cl hydrogen bond. The crystal packing exhibits no short inter­molecular contacts.

## Related literature

For applications of pregnene-type steroidal alkaloid derivatives, see: Hua *et al.* (2005 ▶); Hunter & Priest (2006 ▶). For the crystal structure of the related compound (*Z*)-3α-(1,3-dioxoisoindolin-2-yl)-17 (20)-pregnene, see: Qi *et al.* (2011 ▶).
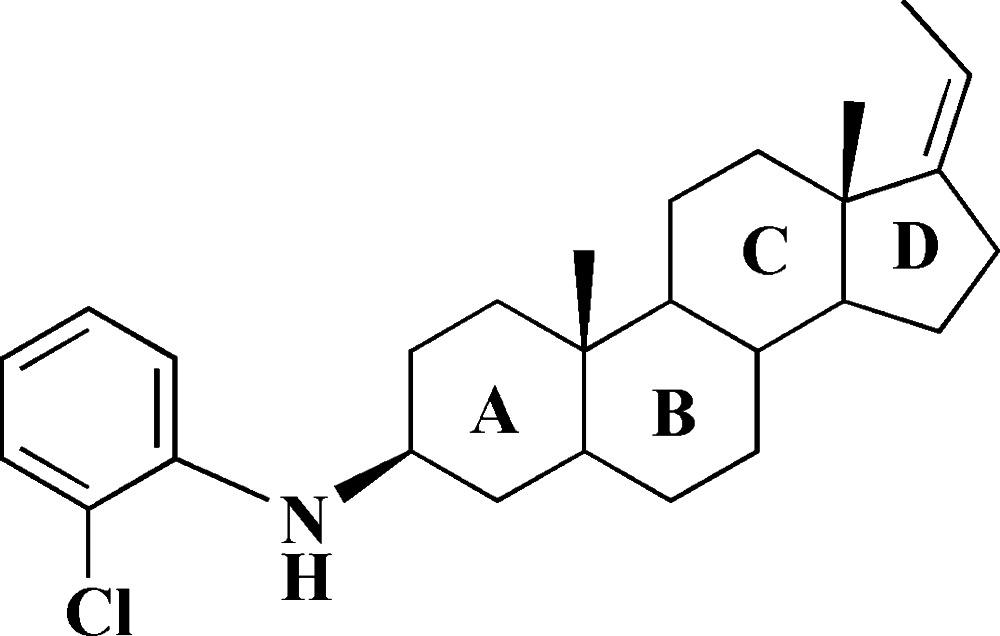


## Experimental

### 

#### Crystal data

C_27_H_38_ClN
*M* *_r_* = 412.03
Monoclinic, 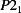

*a* = 7.281 (1) Å
*b* = 17.467 (2) Å
*c* = 9.1310 (12) Å
β = 106.447 (7)°
*V* = 1113.7 (2) Å^3^
*Z* = 2
Mo *K*α radiation
μ = 0.19 mm^−1^
*T* = 113 K
0.26 × 0.24 × 0.18 mm

#### Data collection

Rigaku Saturn724 CCD diffractometer
Absorption correction: multi-scan (*CrystalClear*; Rigaku/MSC, 2005 ▶) *T*_min_ = 0.953, *T*_max_ = 0.967
14303 measured reflections
5276 independent reflections
4089 reflections with *I* > 2σ(*I*)
*R*_int_ = 0.038

#### Refinement

*R*[*F* ^2^ > 2σ(*F* ^2^)] = 0.029
*wR*(*F* ^2^) = 0.066
*S* = 0.97
5276 reflections
269 parameters
1 restraint
H atoms treated by a mixture of independent and constrained refinement
Δρ_max_ = 0.18 e Å^−3^
Δρ_min_ = −0.25 e Å^−3^
Absolute structure: Flack (1983 ▶), 2537 Friedel pairs
Flack parameter: 0.02 (3)

### 

Data collection: *CrystalClear* (Rigaku/MSC, 2005 ▶); cell refinement: *CrystalClear*; data reduction: *CrystalClear*; program(s) used to solve structure: *SHELXS97* (Sheldrick, 2008 ▶); program(s) used to refine structure: *SHELXL97* (Sheldrick, 2008 ▶); molecular graphics: *SHELXTL* (Sheldrick, 2008 ▶); software used to prepare material for publication: *CrystalStructure* (Rigaku/MSC, 2005 ▶).

## Supplementary Material

Crystal structure: contains datablock(s) global, I. DOI: 10.1107/S1600536813007290/cv5384sup1.cif

Structure factors: contains datablock(s) I. DOI: 10.1107/S1600536813007290/cv5384Isup2.hkl

Additional supplementary materials:  crystallographic information; 3D view; checkCIF report

## Figures and Tables

**Table 1:** Hydrogen-bond geometry (Å, °)

*D*—H⋯*A*	*D*—H	H⋯*A*	*D*⋯*A*	*D*—H⋯*A*
N1—H10⋯Cl1	0.875 (19)	2.50 (2)	2.9569 (13)	113.5 (16)

**Fig. 1.**
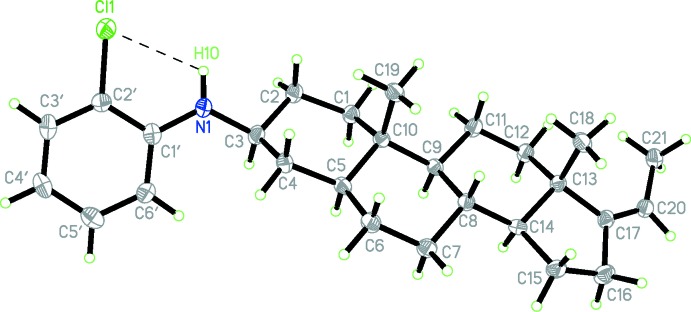
View of (I) showing the atom-labelling scheme. Displacement ellipsoids are drawn at the 55% probability level. Dashed line denotes an intramolecular hydrogen bond.

## supplementary crystallographic information

### Comment

Pregnane-type steroidal alkaloids derivatives are used widely in medicine such as anti-cancer cell, anti-inflammatory and anti-HIV (Hua *et al.*, 2005; Hunter *et al.*, 2006). The goal of evaluating putative small molecule in anti-invasive and anti-metastatic of VEGF receptor selective antagonists has been of great interest in our group. Herewith, we report the synthesis and crystal structure of the title compound, (I).

In (I) (Fig. 1), all bond lengths and angles are normal and correspond to those reported for the related (*Z*)-3α-(1,3-dioxoisoindolin-2-yl)-17 (20)-pregnene (Qi *et al.*, 2011). There are three cyclohexane rings (designated as rings A, B, and C from left to the right) and one cyclopentane ring (the D ring) in a steroid skeleton and hence there are three fusion points. Rings A/B, B/C and C/D all link as *trans*-*trans* -*trans* chair conformation. In the pregnene fragment, three six-membered rings exhibit the chair conformation, and a five-membered ring is in an envelope form. There is an ethenyl group attached to the five-membered ring and the angle of C17—C20—C21 between the ethenyl group and five-membered ring in the pregnane moiety is 129.41 (13)° which is deviated from the normal angle of *sp*^2^ hybridization because of the conformation. 3β-(2-chlorophenyl)amino group is bonded to the pregnene moiety. The 3β-(2-chlorophenyl)amino group is approximately planar, with an r.m.s. deviation 0.0113 (2) Å. The amino group is involved in formation of intramolecular N—H···Cl hydrogen bond (Table 1). The crystal packing exhibits no short intermolecular contacts.

### Experimental

To a suspension of EtPPh_3_Br in anhydrous THF was added t-BuOK. A solution of commercially available epiandrosterone in THF was added. The resultant mixture was refluxed and stirred for 3 h to give pregn-17 (20)-en-3-ol in 50% yield. Steroid pregn-17 (20)-en-3-ol was dissolved in acetone and dichloromethane (1:1.5) then cooled to the temperature of 273 K. Jones reagent was added to give pregn-17 (20) -en-3-one in 55% yield. To a 10% solution of the carbonyl compound pregn-17 (20)-en-3-one in glacial acetic acid was added *o*-chloroaniline with stirring at the temperature of 288 K then sodium borohydride. Upon keeping the reaction mass at room temperature for 2 h afford the title compound (yield 70% after column chromatography purification). Crystals suitable for X-ray structure analysis were recrystalized from petroleum ether/ethyl acetate/dichloromethane (10:1:0.5) by slow evaporation of the solvent at room temperature after several days.

### Refinement

H atom of the amino group was located in a difference map and isotropically refined. Other H atoms were positioned geometrically (C—H = 0.95–1.00 Å) and allowed to ride on their parent atoms, with *U*_iso_(H) = 1.2*U*_eq_ or 1.5*U*_eq_ (parent).

### Crystal data

**Table:**

C_27_H_38_ClN	*F*(000) = 448
*M**_r_* = 412.03	*D*_x_ = 1.229 Mg m^−^^3^
Monoclinic, *P*2_1_	Mo *K*α radiation, λ = 0.71073 Å
Hall symbol: P 2yb	Cell parameters from 4165 reflections
*a* = 7.281 (1) Å	θ = 1.2–27.9°
*b* = 17.467 (2) Å	µ = 0.19 mm^−^^1^
*c* = 9.1310 (12) Å	*T* = 113 K
β = 106.447 (7)°	Prism, colourless
*V* = 1113.7 (2) Å^3^	0.26 × 0.24 × 0.18 mm
*Z* = 2	

### Data collection

**Table:**

Rigaku Saturn724 CCD diffractometer	5276 independent reflections
Radiation source: rotating anode	4089 reflections with *I* > 2σ(*I*)
Multilayer monochromator	*R*_int_ = 0.038
Detector resolution: 14.222 pixels mm^-1^	θ_max_ = 27.9°, θ_min_ = 2.3°
ω scans	*h* = −9→9
Absorption correction: multi-scan (*CrystalClear*; Rigaku/MSC, 2005)	*k* = −22→22
*T*_min_ = 0.953, *T*_max_ = 0.967	*l* = −11→11
14303 measured reflections	

### Refinement

**Table:**

Refinement on *F*^2^	Secondary atom site location: difference Fourier map
Least-squares matrix: full	Hydrogen site location: inferred from neighbouring sites
*R*[*F*^2^ > 2σ(*F*^2^)] = 0.029	H atoms treated by a mixture of independent and constrained refinement
*wR*(*F*^2^) = 0.066	*w* = 1/[σ^2^(*F*_o_^2^) + (0.0299*P*)^2^] where *P* = (*F*_o_^2^ + 2*F*_c_^2^)/3
*S* = 0.97	(Δ/σ)_max_ = 0.001
5276 reflections	Δρ_max_ = 0.18 e Å^−^^3^
269 parameters	Δρ_min_ = −0.25 e Å^−^^3^
1 restraint	Absolute structure: Flack (1983), 2537 Friedel pairs
Primary atom site location: structure-invariant direct methods	Flack parameter: 0.02 (3)

### Special details

**Table:**

Geometry. All e.s.d.'s (except the e.s.d. in the dihedral angle between two l.s. planes) are estimated using the full covariance matrix. The cell e.s.d.'s are taken into account individually in the estimation of e.s.d.'s in distances, angles and torsion angles; correlations between e.s.d.'s in cell parameters are only used when they are defined by crystal symmetry. An approximate (isotropic) treatment of cell e.s.d.'s is used for estimating e.s.d.'s involving l.s. planes.
Refinement. Refinement of *F*^2^ against ALL reflections. The weighted *R*-factor *wR* and goodness of fit *S* are based on *F*^2^, conventional *R*-factors *R* are based on *F*, with *F* set to zero for negative *F*^2^. The threshold expression of *F*^2^ > σ(*F*^2^) is used only for calculating *R*-factors(gt) *etc*. and is not relevant to the choice of reflections for refinement. *R*-factors based on *F*^2^ are statistically about twice as large as those based on *F*, and *R*- factors based on ALL data will be even larger.

### Fractional atomic coordinates and isotropic or equivalent isotropic displacement parameters (Å^2^)

**Table:**

	*x*	*y*	*z*	*U*_iso_*/*U*_eq_	
Cl1	0.65026 (5)	0.47549 (2)	0.27405 (4)	0.02681 (9)	
N1	0.72923 (18)	0.63876 (7)	0.23132 (14)	0.0219 (3)	
C1	0.4738 (2)	0.83125 (8)	0.15203 (16)	0.0184 (3)	
H1A	0.3408	0.8434	0.1515	0.022*	
H1B	0.4885	0.8457	0.0511	0.022*	
C1'	0.86537 (19)	0.59002 (8)	0.20670 (15)	0.0179 (3)	
C2	0.50533 (19)	0.74475 (8)	0.17404 (17)	0.0193 (3)	
H2A	0.4214	0.7179	0.0843	0.023*	
H2B	0.4669	0.7286	0.2650	0.023*	
C2'	0.84853 (19)	0.51050 (8)	0.22294 (15)	0.0188 (3)	
C3	0.7134 (2)	0.72010 (8)	0.19420 (16)	0.0194 (3)	
H3	0.7421	0.7272	0.0943	0.023*	
C3'	0.9773 (2)	0.45928 (8)	0.19481 (17)	0.0235 (3)	
H3'	0.9593	0.4060	0.2064	0.028*	
C4	0.8522 (2)	0.76977 (8)	0.31296 (16)	0.0193 (3)	
H4A	0.8370	0.7586	0.4152	0.023*	
H4B	0.9852	0.7567	0.3149	0.023*	
C4'	1.1333 (2)	0.48461 (9)	0.14969 (15)	0.0260 (3)	
H4'	1.2228	0.4493	0.1302	0.031*	
C5	0.81801 (19)	0.85484 (8)	0.27867 (16)	0.0168 (3)	
H5	0.8293	0.8631	0.1732	0.020*	
C5'	1.1556 (2)	0.56290 (9)	0.13369 (16)	0.0253 (3)	
H5'	1.2617	0.5813	0.1029	0.030*	
C6	0.97282 (19)	0.90405 (8)	0.38521 (17)	0.0213 (3)	
H6A	0.9690	0.8966	0.4918	0.026*	
H6B	1.1002	0.8874	0.3786	0.026*	
C6'	1.0254 (2)	0.61462 (8)	0.16199 (16)	0.0218 (3)	
H6'	1.0446	0.6679	0.1510	0.026*	
C7	0.94543 (18)	0.98860 (8)	0.34445 (16)	0.0203 (3)	
H7A	0.9652	0.9971	0.2428	0.024*	
H7B	1.0425	1.0189	0.4200	0.024*	
C8	0.74540 (18)	1.01621 (7)	0.34212 (16)	0.0156 (3)	
H8	0.7312	1.0116	0.4476	0.019*	
C9	0.58989 (18)	0.96592 (8)	0.23272 (15)	0.0147 (3)	
H9	0.6120	0.9703	0.1298	0.018*	
C10	0.61220 (18)	0.87966 (8)	0.27571 (15)	0.0151 (3)	
C11	0.38697 (19)	0.99738 (7)	0.21507 (16)	0.0197 (3)	
H11A	0.2953	0.9688	0.1323	0.024*	
H11B	0.3527	0.9875	0.3108	0.024*	
C12	0.36389 (19)	1.08327 (8)	0.17908 (17)	0.0192 (3)	
H12A	0.3758	1.0925	0.0752	0.023*	
H12B	0.2344	1.0998	0.1805	0.023*	
C13	0.51445 (19)	1.13112 (7)	0.29440 (15)	0.0151 (3)	
C14	0.71243 (18)	1.09903 (8)	0.29169 (16)	0.0157 (3)	
H14	0.7130	1.1001	0.1823	0.019*	
C15	0.85384 (19)	1.16029 (8)	0.37215 (16)	0.0213 (3)	
H15A	0.9781	1.1549	0.3491	0.026*	
H15B	0.8754	1.1586	0.4841	0.026*	
C16	0.7503 (2)	1.23417 (8)	0.30321 (18)	0.0241 (3)	
H16A	0.7832	1.2766	0.3780	0.029*	
H16B	0.7870	1.2491	0.2107	0.029*	
C17	0.5354 (2)	1.21616 (8)	0.26260 (15)	0.0171 (3)	
C18	0.4833 (2)	1.12587 (8)	0.45464 (16)	0.0217 (3)	
H18A	0.5895	1.1512	0.5293	0.026*	
H18B	0.4779	1.0720	0.4829	0.026*	
H18C	0.3626	1.1511	0.4534	0.026*	
C19	0.57245 (19)	0.86552 (8)	0.43077 (15)	0.0199 (3)	
H19A	0.5888	0.8110	0.4565	0.024*	
H19B	0.4410	0.8810	0.4242	0.024*	
H19C	0.6623	0.8956	0.5101	0.024*	
C20	0.4035 (2)	1.27000 (8)	0.22004 (15)	0.0198 (3)	
H20	0.4507	1.3198	0.2092	0.024*	
C21	0.18870 (19)	1.26393 (8)	0.18609 (17)	0.0228 (3)	
H21A	0.1510	1.2099	0.1759	0.027*	
H21B	0.1274	1.2909	0.0906	0.027*	
H21C	0.1483	1.2871	0.2697	0.027*	
H10	0.635 (3)	0.6182 (12)	0.259 (2)	0.059 (7)*	

### Atomic displacement parameters (Å^2^)

**Table:**

	*U*^11^	*U*^22^	*U*^33^	*U*^12^	*U*^13^	*U*^23^
Cl1	0.02351 (18)	0.01886 (16)	0.0400 (2)	−0.00103 (17)	0.01213 (15)	−0.00106 (17)
N1	0.0254 (7)	0.0144 (6)	0.0291 (7)	0.0032 (5)	0.0132 (6)	0.0030 (5)
C1	0.0165 (7)	0.0165 (7)	0.0206 (8)	0.0016 (6)	0.0030 (6)	0.0003 (6)
C1'	0.0200 (7)	0.0189 (7)	0.0130 (7)	0.0026 (6)	0.0020 (5)	−0.0013 (6)
C2	0.0190 (7)	0.0168 (7)	0.0206 (8)	0.0010 (6)	0.0032 (6)	0.0004 (6)
C2'	0.0175 (7)	0.0200 (7)	0.0180 (7)	−0.0008 (6)	0.0037 (6)	−0.0003 (6)
C3	0.0234 (7)	0.0140 (7)	0.0211 (8)	0.0022 (6)	0.0070 (6)	0.0027 (6)
C3'	0.0263 (8)	0.0176 (8)	0.0239 (8)	0.0050 (6)	0.0026 (6)	−0.0016 (6)
C4	0.0181 (7)	0.0168 (7)	0.0235 (8)	0.0047 (6)	0.0066 (6)	0.0019 (6)
C4'	0.0240 (7)	0.0274 (9)	0.0268 (8)	0.0092 (7)	0.0076 (6)	−0.0013 (7)
C5	0.0152 (7)	0.0182 (7)	0.0176 (7)	0.0025 (5)	0.0056 (6)	0.0010 (6)
C5'	0.0223 (8)	0.0320 (9)	0.0232 (8)	0.0016 (6)	0.0088 (6)	−0.0020 (6)
C6	0.0131 (7)	0.0214 (8)	0.0277 (8)	0.0029 (6)	0.0030 (6)	0.0004 (6)
C6'	0.0240 (8)	0.0175 (8)	0.0241 (8)	0.0002 (6)	0.0069 (6)	0.0015 (6)
C7	0.0130 (6)	0.0196 (9)	0.0265 (8)	0.0002 (5)	0.0029 (6)	0.0003 (6)
C8	0.0129 (7)	0.0156 (7)	0.0177 (7)	0.0003 (5)	0.0033 (5)	−0.0009 (5)
C9	0.0141 (6)	0.0152 (7)	0.0146 (7)	0.0014 (6)	0.0039 (5)	0.0003 (5)
C10	0.0123 (6)	0.0161 (7)	0.0165 (7)	0.0007 (5)	0.0033 (5)	0.0006 (6)
C11	0.0136 (7)	0.0165 (8)	0.0260 (8)	−0.0003 (5)	0.0008 (6)	−0.0006 (6)
C12	0.0140 (7)	0.0159 (7)	0.0253 (8)	0.0012 (5)	0.0018 (6)	−0.0010 (6)
C13	0.0147 (7)	0.0144 (7)	0.0159 (7)	0.0009 (5)	0.0039 (5)	0.0009 (5)
C14	0.0139 (7)	0.0186 (7)	0.0154 (7)	−0.0006 (6)	0.0054 (5)	0.0014 (5)
C15	0.0150 (7)	0.0203 (8)	0.0282 (8)	−0.0018 (6)	0.0052 (6)	−0.0004 (6)
C16	0.0188 (8)	0.0202 (8)	0.0313 (9)	−0.0017 (6)	0.0036 (6)	0.0007 (6)
C17	0.0198 (7)	0.0168 (7)	0.0143 (7)	−0.0019 (6)	0.0043 (6)	−0.0032 (6)
C18	0.0231 (7)	0.0209 (7)	0.0239 (8)	0.0033 (6)	0.0114 (6)	0.0004 (6)
C19	0.0187 (7)	0.0203 (7)	0.0222 (8)	0.0029 (6)	0.0081 (6)	0.0022 (6)
C20	0.0236 (7)	0.0164 (7)	0.0207 (7)	−0.0002 (6)	0.0083 (6)	−0.0006 (6)
C21	0.0249 (8)	0.0184 (8)	0.0266 (8)	0.0032 (6)	0.0099 (6)	−0.0002 (6)

### Geometric parameters (Å, º)

**Table:**

Cl1—C2'	1.7485 (14)	C8—C9	1.5527 (18)
N1—C1'	1.3726 (18)	C8—H8	1.0000
N1—C3	1.4576 (17)	C9—C11	1.5414 (17)
N1—H10	0.875 (19)	C9—C10	1.554 (2)
C1—C2	1.5329 (18)	C9—H9	1.0000
C1—C10	1.5367 (18)	C10—C19	1.5432 (18)
C1—H1A	0.9900	C11—C12	1.5349 (18)
C1—H1B	0.9900	C11—H11A	0.9900
C1'—C2'	1.4060 (19)	C11—H11B	0.9900
C1'—C6'	1.4064 (19)	C12—C13	1.5347 (19)
C2—C3	1.5350 (18)	C12—H12A	0.9900
C2—H2A	0.9900	C12—H12B	0.9900
C2—H2B	0.9900	C13—C17	1.5295 (19)
C2'—C3'	1.3715 (18)	C13—C18	1.5456 (18)
C3—C4	1.5263 (19)	C13—C14	1.5533 (18)
C3—H3	1.0000	C14—C15	1.5219 (19)
C3'—C4'	1.386 (2)	C14—H14	1.0000
C3'—H3'	0.9500	C15—C16	1.537 (2)
C4—C5	1.5244 (19)	C15—H15A	0.9900
C4—H4A	0.9900	C15—H15B	0.9900
C4—H4B	0.9900	C16—C17	1.5355 (19)
C4'—C5'	1.390 (2)	C16—H16A	0.9900
C4'—H4'	0.9500	C16—H16B	0.9900
C5—C6	1.5280 (19)	C17—C20	1.3215 (19)
C5—C10	1.5528 (18)	C18—H18A	0.9800
C5—H5	1.0000	C18—H18B	0.9800
C5'—C6'	1.386 (2)	C18—H18C	0.9800
C5'—H5'	0.9500	C19—H19A	0.9800
C6—C7	1.5222 (19)	C19—H19B	0.9800
C6—H6A	0.9900	C19—H19C	0.9800
C6—H6B	0.9900	C20—C21	1.5092 (19)
C6'—H6'	0.9500	C20—H20	0.9500
C7—C8	1.5286 (18)	C21—H21A	0.9800
C7—H7A	0.9900	C21—H21B	0.9800
C7—H7B	0.9900	C21—H21C	0.9800
C8—C14	1.5166 (18)		
			
C1'—N1—C3	125.43 (13)	C8—C9—C10	112.39 (10)
C1'—N1—H10	117.1 (14)	C11—C9—H9	106.2
C3—N1—H10	116.8 (14)	C8—C9—H9	106.2
C2—C1—C10	113.79 (11)	C10—C9—H9	106.2
C2—C1—H1A	108.8	C1—C10—C19	109.39 (11)
C10—C1—H1A	108.8	C1—C10—C5	106.74 (11)
C2—C1—H1B	108.8	C19—C10—C5	111.72 (10)
C10—C1—H1B	108.8	C1—C10—C9	110.37 (10)
H1A—C1—H1B	107.7	C19—C10—C9	110.70 (11)
N1—C1'—C2'	120.46 (13)	C5—C10—C9	107.84 (10)
N1—C1'—C6'	123.65 (13)	C12—C11—C9	114.48 (11)
C2'—C1'—C6'	115.88 (12)	C12—C11—H11A	108.6
C1—C2—C3	113.46 (11)	C9—C11—H11A	108.6
C1—C2—H2A	108.9	C12—C11—H11B	108.6
C3—C2—H2A	108.9	C9—C11—H11B	108.6
C1—C2—H2B	108.9	H11A—C11—H11B	107.6
C3—C2—H2B	108.9	C13—C12—C11	111.82 (11)
H2A—C2—H2B	107.7	C13—C12—H12A	109.3
C3'—C2'—C1'	122.68 (13)	C11—C12—H12A	109.3
C3'—C2'—Cl1	118.79 (11)	C13—C12—H12B	109.3
C1'—C2'—Cl1	118.48 (10)	C11—C12—H12B	109.3
N1—C3—C4	113.32 (11)	H12A—C12—H12B	107.9
N1—C3—C2	108.16 (11)	C17—C13—C12	119.26 (12)
C4—C3—C2	111.01 (11)	C17—C13—C18	106.65 (11)
N1—C3—H3	108.1	C12—C13—C18	110.45 (12)
C4—C3—H3	108.1	C17—C13—C14	101.81 (11)
C2—C3—H3	108.1	C12—C13—C14	106.17 (11)
C2'—C3'—C4'	120.58 (13)	C18—C13—C14	112.30 (11)
C2'—C3'—H3'	119.7	C8—C14—C15	119.79 (11)
C4'—C3'—H3'	119.7	C8—C14—C13	114.02 (11)
C5—C4—C3	111.79 (11)	C15—C14—C13	104.05 (11)
C5—C4—H4A	109.3	C8—C14—H14	106.0
C3—C4—H4A	109.3	C15—C14—H14	106.0
C5—C4—H4B	109.3	C13—C14—H14	106.0
C3—C4—H4B	109.3	C14—C15—C16	101.91 (11)
H4A—C4—H4B	107.9	C14—C15—H15A	111.4
C3'—C4'—C5'	118.37 (14)	C16—C15—H15A	111.4
C3'—C4'—H4'	120.8	C14—C15—H15B	111.4
C5'—C4'—H4'	120.8	C16—C15—H15B	111.4
C4—C5—C6	111.68 (11)	H15A—C15—H15B	109.3
C4—C5—C10	112.12 (11)	C17—C16—C15	105.97 (11)
C6—C5—C10	112.85 (11)	C17—C16—H16A	110.5
C4—C5—H5	106.6	C15—C16—H16A	110.5
C6—C5—H5	106.6	C17—C16—H16B	110.5
C10—C5—H5	106.6	C15—C16—H16B	110.5
C6'—C5'—C4'	121.03 (14)	H16A—C16—H16B	108.7
C6'—C5'—H5'	119.5	C20—C17—C13	130.08 (13)
C4'—C5'—H5'	119.5	C20—C17—C16	122.11 (13)
C7—C6—C5	111.61 (11)	C13—C17—C16	107.61 (11)
C7—C6—H6A	109.3	C13—C18—H18A	109.5
C5—C6—H6A	109.3	C13—C18—H18B	109.5
C7—C6—H6B	109.3	H18A—C18—H18B	109.5
C5—C6—H6B	109.3	C13—C18—H18C	109.5
H6A—C6—H6B	108.0	H18A—C18—H18C	109.5
C5'—C6'—C1'	121.45 (14)	H18B—C18—H18C	109.5
C5'—C6'—H6'	119.3	C10—C19—H19A	109.5
C1'—C6'—H6'	119.3	C10—C19—H19B	109.5
C6—C7—C8	111.58 (11)	H19A—C19—H19B	109.5
C6—C7—H7A	109.3	C10—C19—H19C	109.5
C8—C7—H7A	109.3	H19A—C19—H19C	109.5
C6—C7—H7B	109.3	H19B—C19—H19C	109.5
C8—C7—H7B	109.3	C17—C20—C21	129.41 (13)
H7A—C7—H7B	108.0	C17—C20—H20	115.3
C14—C8—C7	111.92 (11)	C21—C20—H20	115.3
C14—C8—C9	108.69 (11)	C20—C21—H21A	109.5
C7—C8—C9	110.42 (11)	C20—C21—H21B	109.5
C14—C8—H8	108.6	H21A—C21—H21B	109.5
C7—C8—H8	108.6	C20—C21—H21C	109.5
C9—C8—H8	108.6	H21A—C21—H21C	109.5
C11—C9—C8	111.62 (11)	H21B—C21—H21C	109.5
C11—C9—C10	113.61 (10)		
			
C3—N1—C1'—C2'	−169.28 (13)	C4—C5—C10—C9	−177.45 (11)
C3—N1—C1'—C6'	9.5 (2)	C6—C5—C10—C9	55.39 (14)
C10—C1—C2—C3	−52.24 (16)	C11—C9—C10—C1	59.98 (14)
N1—C1'—C2'—C3'	177.82 (13)	C8—C9—C10—C1	−172.07 (10)
C6'—C1'—C2'—C3'	−1.1 (2)	C11—C9—C10—C19	−61.28 (13)
N1—C1'—C2'—Cl1	0.32 (18)	C8—C9—C10—C19	66.67 (13)
C6'—C1'—C2'—Cl1	−178.57 (10)	C11—C9—C10—C5	176.22 (11)
C1'—N1—C3—C4	−80.43 (17)	C8—C9—C10—C5	−55.83 (13)
C1'—N1—C3—C2	156.05 (13)	C8—C9—C11—C12	49.27 (15)
C1—C2—C3—N1	173.59 (11)	C10—C9—C11—C12	177.62 (11)
C1—C2—C3—C4	48.69 (16)	C9—C11—C12—C13	−52.96 (16)
C1'—C2'—C3'—C4'	0.6 (2)	C11—C12—C13—C17	170.00 (11)
Cl1—C2'—C3'—C4'	178.12 (11)	C11—C12—C13—C18	−65.98 (15)
N1—C3—C4—C5	−174.30 (11)	C11—C12—C13—C14	56.00 (15)
C2—C3—C4—C5	−52.36 (16)	C7—C8—C14—C15	−53.21 (16)
C2'—C3'—C4'—C5'	−0.1 (2)	C9—C8—C14—C15	−175.43 (11)
C3—C4—C5—C6	−172.62 (11)	C7—C8—C14—C13	−177.36 (11)
C3—C4—C5—C10	59.60 (15)	C9—C8—C14—C13	60.42 (14)
C3'—C4'—C5'—C6'	0.0 (2)	C17—C13—C14—C8	171.90 (11)
C4—C5—C6—C7	176.45 (11)	C12—C13—C14—C8	−62.61 (14)
C10—C5—C6—C7	−56.16 (15)	C18—C13—C14—C8	58.19 (15)
C4'—C5'—C6'—C1'	−0.5 (2)	C17—C13—C14—C15	39.67 (13)
N1—C1'—C6'—C5'	−177.85 (13)	C12—C13—C14—C15	165.16 (11)
C2'—C1'—C6'—C5'	1.0 (2)	C18—C13—C14—C15	−74.04 (14)
C5—C6—C7—C8	55.07 (16)	C8—C14—C15—C16	−171.63 (12)
C6—C7—C8—C14	−176.32 (11)	C13—C14—C15—C16	−42.82 (13)
C6—C7—C8—C9	−55.10 (15)	C14—C15—C16—C17	29.17 (15)
C14—C8—C9—C11	−50.99 (14)	C12—C13—C17—C20	47.9 (2)
C7—C8—C9—C11	−174.11 (11)	C18—C13—C17—C20	−77.94 (18)
C14—C8—C9—C10	−179.98 (10)	C14—C13—C17—C20	164.22 (14)
C7—C8—C9—C10	56.89 (14)	C12—C13—C17—C16	−137.25 (13)
C2—C1—C10—C19	−65.95 (15)	C18—C13—C17—C16	96.90 (13)
C2—C1—C10—C5	55.09 (14)	C14—C13—C17—C16	−20.94 (14)
C2—C1—C10—C9	172.02 (11)	C15—C16—C17—C20	170.51 (13)
C4—C5—C10—C1	−58.86 (13)	C15—C16—C17—C13	−4.83 (16)
C6—C5—C10—C1	173.99 (11)	C13—C17—C20—C21	−1.4 (2)
C4—C5—C10—C19	60.68 (15)	C16—C17—C20—C21	−175.62 (14)
C6—C5—C10—C19	−66.48 (14)		

### Hydrogen-bond geometry (Å, º)

**Table:**

*D*—H···*A*	*D*—H	H···*A*	*D*···*A*	*D*—H···*A*
N1—H10···Cl1	0.875 (19)	2.50 (2)	2.9569 (13)	113.5 (16)
